# Assembled Au/ZnO Nano-Urchins for SERS Sensing of the Pesticide Thiram

**DOI:** 10.3390/nano11092174

**Published:** 2021-08-25

**Authors:** Grégory Barbillon, Octavio Graniel, Mikhael Bechelany

**Affiliations:** 1 EPF-Ecole d’Ingénieurs, 3 Bis Rue Lakanal, 92330 Sceaux, France; 2 Institut Européen des Membranes (IEM), UMR-5635, Université de Montpellier, ENSCM, CNRS, Place Eugène Bataillon, 34095 Montpellier, France; ograniel@gmail.com (O.G.); mikhael.bechelany@umontpellier.fr (M.B.)

**Keywords:** SERS, sensors, plasmonics, gold, zinc oxide, thiram, adsorption

## Abstract

In this paper, we are relating a significant improvement of the SERS effect achieved with assembled Au/ZnO nano-urchins. This improvement is realized thanks to an excellent capacity of adsorption (denoted *K*) for thiram molecules on these plasmonic nano-urchins, which is a key point to be taken into account for obtaining a SERS spectrum. Moreover, this outlook may be employed for different types of plasmonic substrates and for a wide number of molecules. We studied the capacity of the assembled Au/ZnO nano-urchins to be sensitive to the pesticide thiram, which adsorbs well on metals via the metal–sulfur bond. For the thiram detection, we found a limit concentration of 10 pM, a value of this capacity of adsorption (*K*) of 9.5 × 10${}^{6}$ M${}^{-1}$ and a factor of analytical enhancement equal to 1.9 × 10${}^{8}$.

## 1. Introduction

Thiram is a sulfur-containing pesticide that is broadly employed in agriculture as a protective agent for the production of vegetables and fruits [1,2]. Moreover, thiram is also employed in soaps as bactericide and in the rubber industry [3]. In addition, the exposure to thiram can engender various diseases or other health problems due to the liberation of the carbon disulfide compound [4,5,6,7]. Moreover, thiram can also contaminate surface and groundwater due to its transportation by diffuse pathways. During the last ten years, different detection techniques have been employed for the detection of thiram, such as fluorescence [8], colorimetry [9], electrochemistry [10], resonance Rayleigh scattering [11], plasmonic absorption [3], and the localized surface plasmon resonance [12]. With these techniques, the detection limits of the pesticide thiram vary between 10${}^{-9}$ M to 10${}^{-6}$ M. In addition, another technique is also used, which is the surface-enhanced Raman scattering (SERS). This technique consists in the use of plasmonic or hybrid (metal/semiconductor) nanostructures/nanoparticles [13,14,15,16,17,18,19] in order to improve the SERS signal of biochemical analytes via electric fields of the plasmonic or hybrid nanostructures/nanoparticles [20]. These plasmonic nanostructures/nanoparticles can be fabricated by chemical synthesis [21,22,23,24] or lithographic techniques [25,26,27,28,29,30,31,32]. Moreover, several groups have already investigated plasmonic or hybrid nanostructures/nanoparticles for thiram detection by SERS with detection limits varying from 10${}^{-8}$ M to 10${}^{-7}$ M [33,34,35,36,37]. Nevertheless, in these different investigations, this capacity of adsorption (*K*) has been not considered for obtaining a SERS spectrum. The Langmuir model allows determining this adsorption constant (denoted *K*) [38,39,40,41].

The goal of this investigation is to exhibit a highly sensitive detection of thiram (model pesticide) by using assembled Au/ZnO nano-urchins. This type of nanostructures will permit producing: (i) huge electric fields inside nanogaps between the branches of one same nano-urchin and also between each nano-urchin, and (ii) an excellent capacity of adsorption for thiram on the gold part of these assembled nano-urchins.

## 2. Experimental Details

### 2.1. Fabrication of the Assembled Au/ZnO Nano-Urchins

Au/ZnO urchin-like nanostructures are fabricated following our previously established method (see reference [42]). Firstly, quartz substrates (1 × 2 cm${}^{2}$) covered with indium tin oxide (ITO) are cleaned in a sequential fashion for a duration of 15 min in acetone, then ethanol, and finally isopropanol. Lastly, the ITO/quartz substrates are dried in a stream of air. After, a glass slide (25 × 75 mm${}^{2}$) is rendered hydrophilic with an oxygen plasma treatment and is placed on the vertical edge of a Petri dish, which is full of deionised (DI) water. We carefully add a solution composed of polystyrene (PS) spheres (1:2 PS spheres/anhydrous ethanol) via a micropipette onto the slanted glass substrate until all of the water surface is covered with PS spheres. Hereafter, we add a small volume (5 µL) of a sodium dodecyl sulfate solution to the surface of water for promoting the tight packing of the PS spheres. An ITO/quartz substrate (rendered hydrophilic by exposing it to an UV light for a duration of 15 min) is then introduced into the Petri dish at an angle of 45${}^{\circ }$ to transfer PS spheres towards the substrate. Once the substrates were dry, they are introduced for 30 min in an oven set at a temperature of 100 ${}^{\circ }$C to favor the sphere adhesion. Next, PS spheres are exposed to an O${}_{2}$ plasma treatment to reduce their dimension and form a non-close-packed arrangement. Afterwards, a 20 nm thin film of ZnO is deposited on the PS spheres substrates with a home-made apparatus of atomic layer deposition (ALD). The deposition is carried at 80 ${}^{\circ }$C with diethylzinc (DEZ) and H${}_{2}$O as precursors. Subsequently, ZnO nanowires are grown on the ZnO-covered PS spheres with a three-electrode configuration consisting of the ZnO/PS spheres/ITO/quartz substrate (working electrode), a Pt sheet of 1 × 1 cm${}^{2}$ serving as counter-electrode, and an Ag/AgCl electrode serving as reference. These latter are introduced into an electrolyte solution composed of ZnCl${}_{2}$ (0.05 mM) and KCl (0.1 M) and continuously bubbled with O${}_{2}$ during the whole deposition. A constant electric potential of −1 V is applied for a duration of 15 min in a potentiostat (VersaSTAT 3, Princeton Applied Research, Berwyn, PA, USA) and the electrodeposition cell is kept at a temperature of 80 ${}^{\circ }$C. Once the electrodeposition was finished, the PS spheres are burned away from the substrate in an oven (temperature = 600 ${}^{\circ }$C) for a duration of 2 h in air. Lastly, a gold layer with a thickness of 50 nm is deposited with an electron beam evaporation equipment (350 UNIVEX, Oerlikon Leybold, Cologne, Germany) running at a voltage of 10 kV and a pressure of 10${}^{-6}$ mbar. A deposition rate of 0.3 nm s${}^{-1}$ was measured in situ with a quartz crystal microbalance (QCM) monitor. This 50 nm Au layer allows the best enhancement of the SERS signal with our assembled Au/ZnO urchins [42]. The morphology analysis of Au/ZnO nano-urchins has been realized with a scanning electron microscope (Hitachi S-4800, Tokyo, Japan). The structural characterization of assembled hybrid nano-urchins was realized with a diffractometer (PANalytical X’pert-PRO, PANalytical, Malvern, United Kingdom) equipped with an X’celerator detector, which employs a Ni-filtered Cu Kα radiation.

### 2.2. Thiram Functionalization on Assembled Au/ZnO Nano-Urchins

To investigate the sensitivity of the assembled Au/ZnO nano-urchins, we employ the pesticide thiram for its capacity to be grafted on the metallic surface via the gold-sulfur bond [43,44,45]. In the first place, we realized thiram solutions (thiram + ethanol) whose the concentrations were comprised between 10 pM and 1 mM. Next, we immersed the substrates in the thiram solution for a duration of 24 h. Afterwards, the substrates were thoroughly washed with pure ethanol to remove unbound thiram. After this step of washing, we thoroughly dried the assembled Au/ZnO nano-urchins by using a nitrogen gun. In addition, we employed a thiram concentration of 1 mM for the reference experiment.

### 2.3. Raman Experiments

Concerning the Raman experiments, we employ a spectrometer (Labram, Horiba Scientific, Kyoto, Japan) whose the spectral resolution is 1 cm${}^{-1}$. We choose a wavelength of 785 nm for excitation, which allows the best enhancement of the SERS signal with our assembled Au/ZnO urchins [42]. We adjust the acquisition time at 10 s and the laser power at 3 mW. Concerning to the SERS experiments, the laser beam is focused on the plasmonic nano-urchins by a microscope objective (×100, N.A. = 0.9), then the SERS signal of the pesticide thiram is collected by this same way. In addition, for the reference experiment (a 50 nm-thick gold film without any ZnO nano-urchins), the same excitation wavelength, acquisition time, and laser power were used.

## 3. Results and Discussion

**Figure 1:**
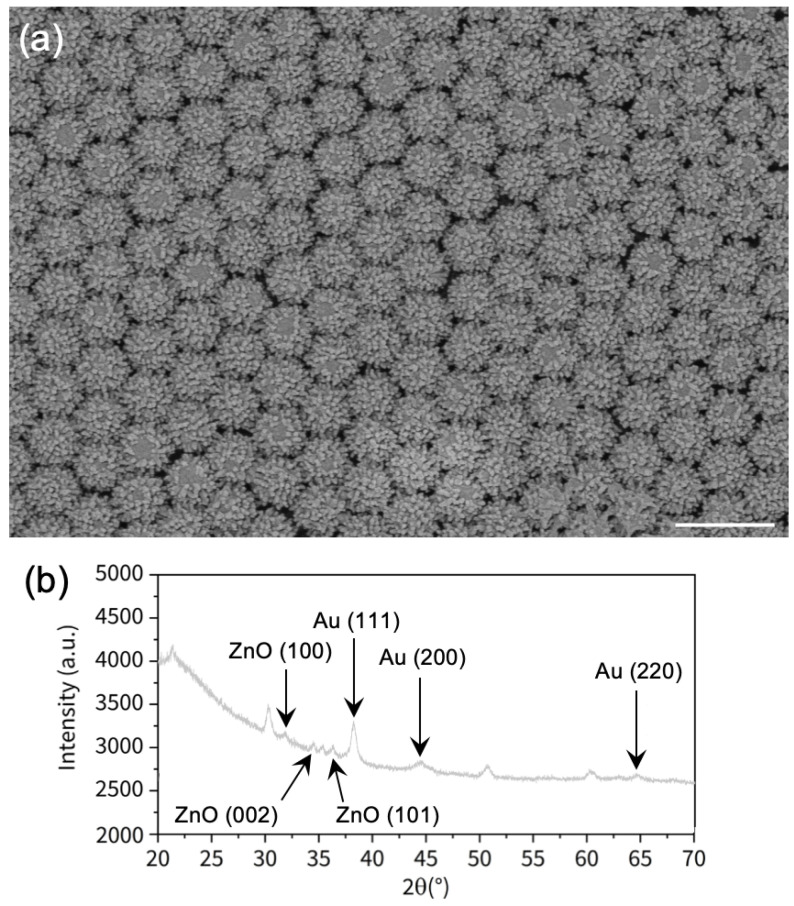
(**a**) SEM image of assembled zinc oxide nano-urchins covered by the 50 nm-thick Au layer (scale bar = 1000 nm). (**b**) XRD pattern of Au/ZnO nano-urchins.

The assembled Au/ZnO nano-urchins were fabricated following the process detailed in Section 2.1, and a SEM image of these hybrid nano-urchins is presented in the Figure 1a. Figure 1b displays the X-ray diffraction (XRD) pattern of our hybrid nano-urchins. The XRD peaks located at 31.8${}^{\circ }$, 34.4${}^{\circ }$, and 36.4${}^{\circ }$ correspond to the (100), (002), and (101) planes of ZnO (hexagonal wurtzite), respectively. The XRD peaks located at 38.2${}^{\circ }$, 44.5${}^{\circ }$, and 64.6${}^{\circ }$ are assigned to the (111), (200), and (220) planes of Au (face-centered-cubic), respectively. Thus, all these XRD peaks successfully confirm the composite nature of our Au/ZnO nano-urchins. The other XRD peaks are assigned to the ITO/quartz substrate.

**Figure 2:**
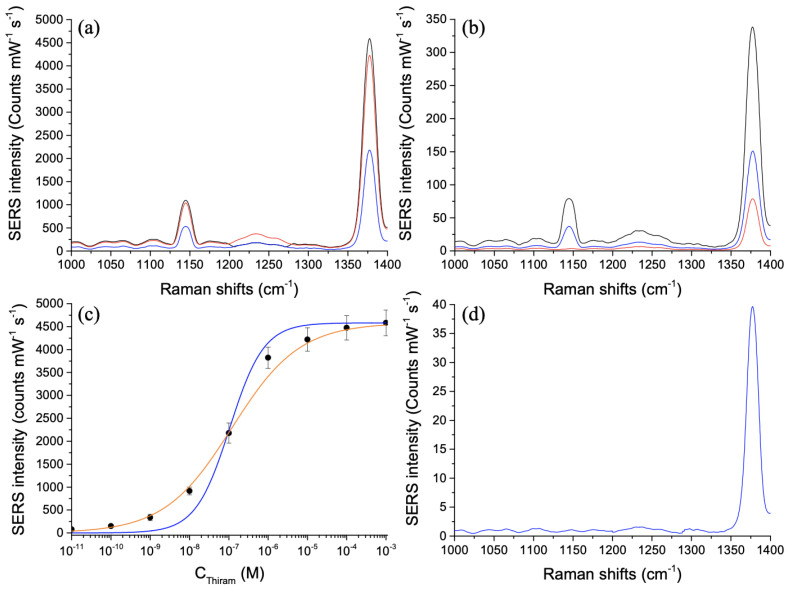
SERS spectra of the pesticide thiram recorded on assembled hybrid nano-urchins: (**a**) in black, C${}_{Thiram}$ = 10${}^{-3}$ M; in red, C${}_{Thiram}$ = 10${}^{-5}$ M, and in blue, C${}_{Thiram}$ = 10${}^{-7}$ M; (**b**) in black, C${}_{Thiram}$ = 10${}^{-9}$ M; in blue, C${}_{Thiram}$ = 10${}^{-10}$ M, and in red, C${}_{Thiram}$ = 10${}^{-11}$ M. (**c**) SERS intensity as function of C${}_{Thiram}$ (=$C_{S}$). The black circles represent the experimental points. The blue curve represents the fitting curve with the expression of the Langmuir model. The orange curve represents the fitting curve with the expression of the Hill model. (**d**) SERS spectrum of the pesticide thiram on a 50 nm-thick gold film (without any ZnO nano-urchins) for a thiram concentration of 1 mM serving as reference.

In order to evaluate the capacity of Au/ZnO nano-urchins to be sensitive, thiram molecules were deposited on the nano-urchins by using the protocol detailed in the Section 2.2. Hereafter, we realized SERS spectra of thiram on hybrid nano-urchins for the wavelength of excitation of 785 nm, and Figure 2 shows these latter. We have chosen this excitation wavelength of 785 nm, because, in a previous work [42], we have demonstrated a better enhancement of the SERS signal for a gold layer of 50 nm-thick and this excitation wavelength of 785 nm. Indeed, in this work, we have studied the SERS activity for three thicknesses (10 nm, 30 nm, and 50 nm) of the gold layer deposited on ZnO nano-urchins and for two excitation wavelengths (633 nm and 785 nm). We found the best SERS signal obtained with the following parameter couple: a thickness of 50 nm for the gold layer and an excitation wavelength of 785 nm. Thus, for this parameter couple, the enhancement of the SERS signal can be remarkably improved due to the fact that this excitation wavelength is nearer one plasmonic resonance for the thickest gold layer (see [42] for more details). From the Figure 2a,b, two Raman peaks of thiram molecules [45,46,47] are well-distinguished: the one at 1145 cm${}^{-1}$ is attributed to the mode of C–N stretching (called: ν(CN)) and the mode of $CH_{3}$ rocking, and the one at 1378 cm${}^{-1}$ corresponds to the mode of C–N stretching (called: ν(CN)) and the mode of symmetric $CH_{3}$ deformation (called: $\delta_{s}\left(CH_{3}\right)$). We have chosen the vibration mode at 1378 cm${}^{-1}$ for its strongest SERS intensity for assessing the capacity of Au/ZnO nano-urchins to be sensitive. We plot the SERS intensity versus thiram concentration, as shown in Figure 2c. From Figure 2b,c, we found a limit concentration (LOD) of 10 pM with a signal-to-noise ratio (S/N) > 3. For thiram concentrations below 10 pM, no Raman peak is discernible (S/N ≪ 3).

Moreover, this LOD is more sensitive than those obtained with other geometries of nanostructures, such as Ag dendritic nanostructures (LOD = 10${}^{-7}$ M, [48]), Ag nanoparticles on a flexible film (LOD = 10${}^{-7}$ M, [49]), Ag nanowires (LOD = 10${}^{-7}$ M, [50]), Ag nanoshells (LOD = 10${}^{-8}$ M, [51]), Au@Ag nanoparticles (LOD = 8 × 10${}^{-11}$–10${}^{-7}$ M, [52,53,54]), Ag/CeO${}_{2}$ nanostructures (LOD = 10${}^{-9}$ M, [47]), and Au nanostars with fractal structure (LOD = 10${}^{-10}$ M, [45]). In addition, the experimental results are fitted by using a Langmuir model [38,39,40,41]:

(1) $$\begin{array}{c} I=I_{max}\frac{KC_{S}}{1+KC_{S}} \end{array}$$

where C${}_{S}$ is the thiram solution concentration. *K* represents the capacity of adsorption (adsorption constant). $I_{max}$ depicts the strongest SERS intensity when the thiram monolayer is completely formed, and *I* is the SERS intensity associated with C${}_{S}$. We note a nice accordance between experimental points and the fitting curve calculated with the expression of a Langmuir model, as shown in Figure 2c. Since the extraction of *K* of the blue curve, we find *K* = 9.5 × 10${}^{6}$ M${}^{-1}$. This latter points out a nice affinity of adsorption for the pesticide thiram on the Au portion of assembled Au/ZnO nano-urchins. Moreover, the experimental results are fitted by employing another model named Hill equation [55,56,57]:

(2) $$\begin{array}{c} I=I_{max}\frac{1}{1+{\left(\frac{k}{C_{S}}\right)}^{n}} \end{array}$$

where *I*, $I_{max}$ and C${}_{S}$ are parameters identical to the previous model. *n* represents the coefficient of Hill. *k* corresponds to the analyte concentration that produces a coverage of 50%. We observe an excellent accordance between experimental points and the fitting curve calculated with the expression of the Hill model, as shown in Figure 2c. Since the extraction of *k* and *n* of the orange curve, we find *k* = 1.2 × 10${}^{-7}$ M and *n* = 0.51. This value of 1.2 × 10${}^{-7}$ M for *k* corroborates the large affinity of thiram regarding the metal surface. Nonetheless, the obtained *n* is less than 1, which indicates a decrease in the affinity of the thiram molecules moving towards the metal surface that already has thiram molecules grafted to its surface [55]. Finally, we calculate the analytical enhancement factor (AEF) of the plasmonic nano-urchins at LOD, with the following expression [58]:

(3) $$\begin{array}{c} AEF=\frac{I_{SERS}}{I_{Raman}}\times \frac{C_{Raman}}{C_{SERS}} \end{array}$$

where $C_{SERS}$ (10 pM) is the thiram concentration for SERS experiment, and $C_{Raman}$ (1 mM) is the thiram concentration for reference experiment. $I_{SERS}$ (see the red SERS spectrum in Figure 2b) is the SERS intensity, and $I_{Raman}$ (see the blue SERS spectrum in Figure 2d) is the Raman intensity. We obtain an AEF value of 1.9 × 10${}^{8}$ (see Table 1).

To finish this investigation, we demonstrated a good uniformity of the SERS signal obtained with assembled Au/ZnO nano-urchins over the whole substrate. For each experimental point of the Figure 2c, SERS spectra have been recorded from ten randomly chosen positions on the sample with identical conditions of excitation for the calculation of the relative standard deviation (RSD).

**Figure 3:**
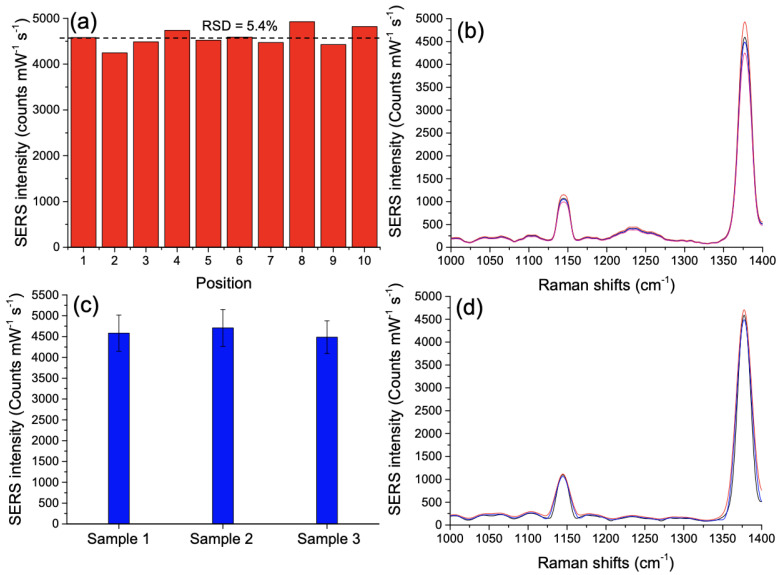
(**a**) SERS intensity recorded for the Raman peak at 1378 cm${}^{-1}$ at ten positions taken randomly on the substrate (the black dashed line represents the average value of the SERS intensity with a RSD value of 5.4%). (**b**) SERS spectra for four positions of (**a**): in red at the position 8, in black at the position 6, in blue at the position 3 and in pink at the position 2. (**c**) SERS intensity distribution of the 1378 cm${}^{-1}$ peak for three distinct samples with an average RSD < 9%. (**d**) SERS spectra associated to three samples: in red for the sample 2, in black for the sample 1 and in blue for the sample 3.

Figure 3a shows the SERS intensity of the Raman peak at 1378 cm${}^{-1}$ recorded at ten positions taken randomly on the substrate (RSD = 5.4%) for the thiram concentration of 1 mM, and Figure 3b displays SERS spectra obtained from four completely opposite positions on the substrate (for other thiram concentrations, see Figures S1 and S2 in Supplementary Information). As for the uniformity of the SERS signal on the whole sample, the sample-to-sample reproducibility was studied by measuring the SERS intensity of the same Raman peak (1378 cm${}^{-1}$) from three distinct samples. Figure 3c shows the SERS intensity distribution of these three distinct samples for the thiram concentration of 1 mM with an average RSD < 9%, and Figure 3d represents the SERS spectra associated to three distinct samples. Thus, we observed a good uniformity and reproducibility of the SERS signal for our assembled hybrid nano-urchins with an average RSD < 9%.

## 4. Conclusions

We have shown the strong capacity of these assembled Au/ZnO nano-urchins to be sensitive to the pesticide thiram. This latter adsorbs well on metals via the metal–sulfur bond, and serves mainly as protective agent in agricultural production of vegetables and fruits. For this detection, we obtained a LOD of 10 pM, a *K* of 9.5 × 10${}^{6}$ M${}^{-1}$, and an AEF of 1.9 × 10${}^{8}$. This LOD of 10 pM was more sensitive than those obtained elsewhere on the plasmonic nanostructures cited above in this paper. We also demonstrated a good uniformity and reproducibility of the SERS signal for our assembled hybrid nano-urchins with an average RSD < 9%, and that the adsorption capacity of the pesticide thiram on Au surface was excellent thanks to the obtaining of a large value for *K*. Furthermore, this high adsorption capacity of the pesticide thiram on Au surface of assembled nano-urchins was corroborated by a value of 1.2 × 10${}^{-7}$ M for *k*. Finally, this approach can be employed for different types of SERS substrate and for a wide number of molecules. Thus, it is important to take into account the capacity of adsorption of molecules on the metal surface in order to reach the highest value of SERS signal in addition to the usual considerations (as the conception of plasmonic/hybrid nanostructures/nanoparticles).

## Figures and Tables

**Table 1:** Parameters for the calculation of AEF using Equation (3).

Parameter Designation	Parameter Values
Vibration mode (cm^−1^)	1378
$I_{SERS}$ (counts mW${}^{-1}$ s${}^{-1}$)	77
$I_{Raman}$ (counts mW${}^{-1}$ s${}^{-1}$)	40
$C_{Raman}$ (M)	10${}^{-3}$
$C_{SERS}$ (M)	10${}^{-11}$

## Data Availability

Data is contained within the article.

## Supplementary Materials

The following are available online at https://www.mdpi.com/article/10.3390/nano11092174/s1, Figure S1: SERS intensity of thiram recorded for the Raman peak at 1378 cm${}^{-1}$, for the concentrations from 10${}^{-4}$ M to 10${}^{-11}$ M at ten positions taken randomly on the substrate, Figure S2: SERS spectra of thiram for the concentrations from 10${}^{-4}$ M to 10${}^{-11}$ M at two of ten positions taken randomly on the substrate.
