# Supplementary material for: Assembled Au/ZnO Nano-Urchins for SERS Sensing of the Pesticide Thiram
Source: Nanomaterials (Basel). 2021 Aug 25;11(9):2174. doi: 10.3390/nano11092174 (PMC8467743; doi:10.3390/nano11092174)
Supplement: Supplementary file 1 [file nanomaterials-11-02174-s001.zip › nanomaterials-1334576-supplementary new.pdf]

Supplementary Information

Assembled Au/ZnO Nano-Urchins for SERS Sensing of the Pesticide Thiram

Grégory Barbillon[1,*] , Octavio Graniel[2] and Mikhael Bechelany[2]

[1]EPF-École d'Ingénieurs, 3 bis rue Lakanal, 92330 Sceaux, France
[2]Institut Européen des Membranes (IEM), UMR-5635, Université de Montpellier, ENSCM, CNRS, Place Eugène Bataillon, 34095 Montpellier, France; ograniel@gmail.com (O.G.); mikhael.bechelany@umontpellier.fr (M.B.)
*****Correspondence: gregory.barbillon@epf.fr

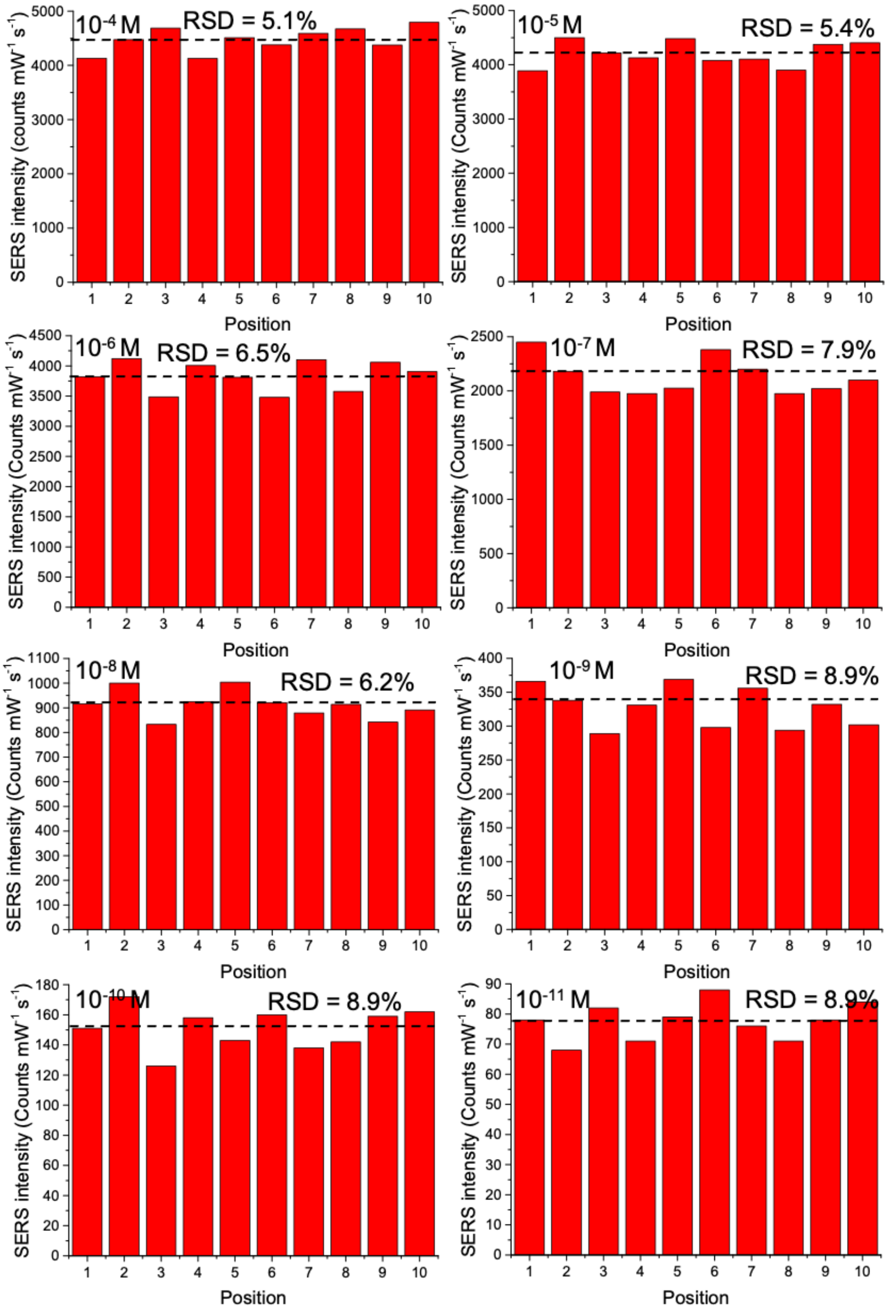

Figure S1. SERS intensity of thiram recorded for the Raman peak at 1378 cm^{-1}, for the concentrations from 10^{-4} M to 10^{-11} M at ten positions taken randomly on the substrate (the black dashed line represents the average value of the SERS intensity and the RSD values are also indicated).

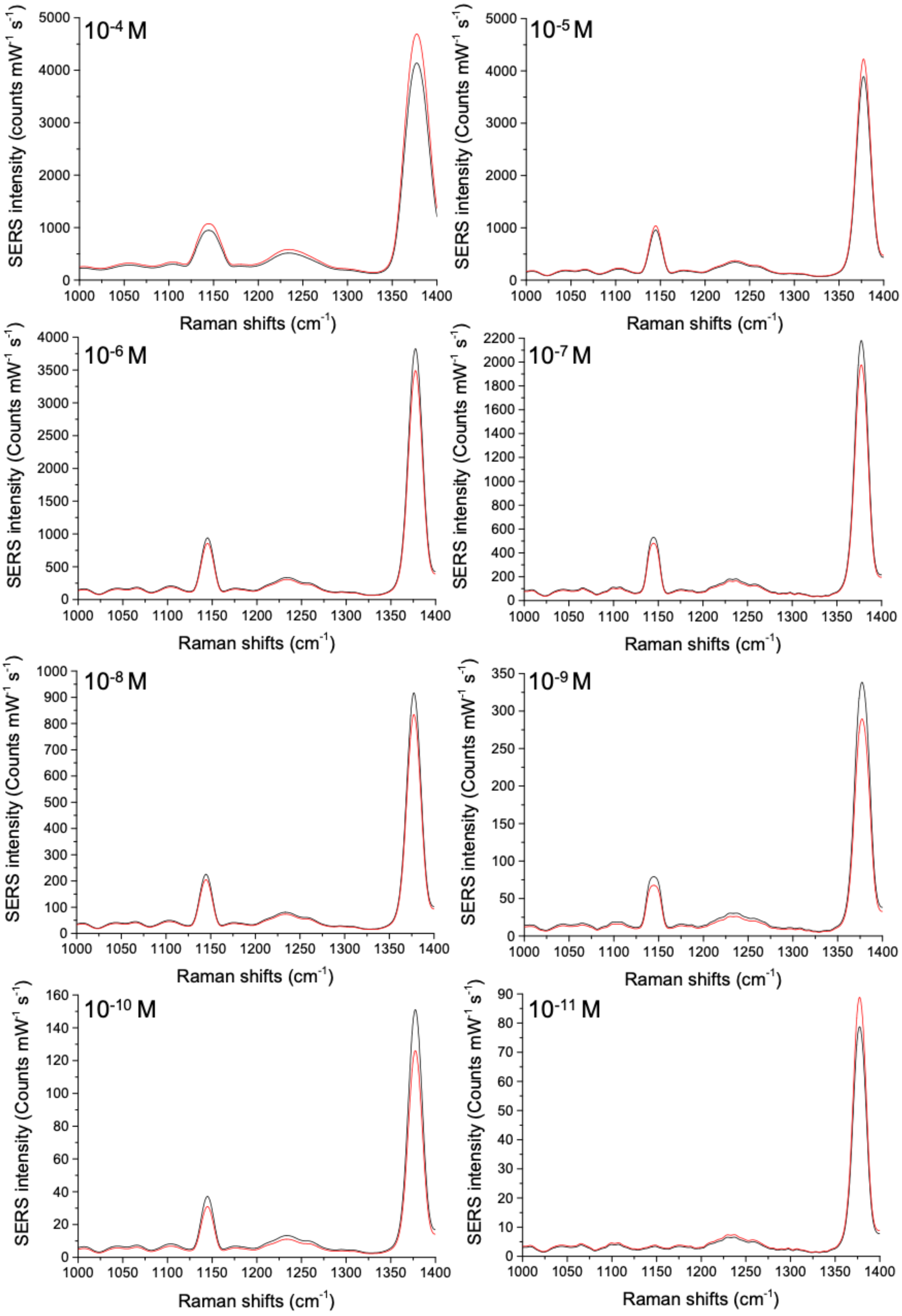

Figure S2. SERS spectra of thiram recorded for the concentrations from 10^{-4} M to 10^{-11} M at two of ten positions taken randomly on the substrate.
